# Views of European Drug Development Stakeholders on Treatment Optimization and Its Potential for Use in Decision-Making

**DOI:** 10.3389/fphar.2020.00043

**Published:** 2020-02-05

**Authors:** Robbe Saesen, Stéphane Lejeune, Gianluca Quaglio, Denis Lacombe, Isabelle Huys

**Affiliations:** ^1^ European Organisation for Research and Treatment of Cancer, Brussels, Belgium; ^2^ Department of Pharmaceutical and Pharmacological Sciences, Clinical Pharmacology and Pharmacotherapy, Katholieke Universiteit Leuven, Leuven, Belgium; ^3^ Panel for the Future of Science and Technology, European Parliamentary Research Service, Brussels, Belgium

**Keywords:** drug development, clinical research, real-world evidence, treatment optimization, qualitative research, health technology assessment, regulatory science, pharmaceutical industry

## Abstract

**Background:**

The current drug development paradigm has been criticized for being too drug-centered and for not adequately focusing on the patients who will eventually be administered the therapeutic interventions it generates. The drug-driven nature of the present framework has led to the emergence of a research gap between the pre-approval development of anticancer medicines and their post-registration use in real-life clinical practice. This gap could potentially be bridged by transitioning toward a patient-centered paradigm that places a strong emphasis on treatment optimization, which strives to optimize the way health technologies are applied in a real-world environment. However, questions remain concerning the ideal features of treatment optimization studies and their acceptability among key stakeholders.

**Objectives:**

The aim of this study was to explore the views of key stakeholders in the drug development process regarding the concept of treatment optimization.

**Methods:**

Semi-structured interviews were conducted between December 2018 and May 2019 with 26 participants across ten EU Member States and six different stakeholder groups, including academic clinicians as well as representatives of patient organizations, regulatory authorities, health technology assessment agencies, payers, and industry.

**Results:**

Based on the input of the experts interviewed, clarification was obtained regarding the optimal features of treatment optimization studies in terms of their conduct, funding, timing, design, and setting. Moreover, a number of opportunities and challenges of undertaking such trials were identified. Inter-stakeholder discussion during their design was seen as desirable. There was also broad support among the participants for regulatory measures to facilitate treatment optimization, although there was no agreement on the optimal scale and nature of these initiatives. Furthermore, the interviewees believed that the evidence strength of well-designed treatment optimization studies performed according to rigorous quality standards is greater than or at least equal to that of classical clinical trials. In addition, there was a strong consensus that the results of treatment optimization studies should be taken into account during the decision-making of regulators, payers, and/or clinicians.

**Conclusions:**

Stakeholders involved in drug development consider treatment optimization studies to be valuable tools to address current evidence gaps and support their implementation into the existing research framework.

## Introduction

The current clinical drug development framework has generated many innovative medicines whose safety, quality, and efficacy have been demonstrated by the manufacturer and evaluated by the competent regulatory authorities. In the European Union, the Committee for Human Medicinal Products of the European Medicines Agency (EMA) assesses registration dossiers submitted by pharmaceutical companies for health technologies that are subject to the so-called centralized procedure, which, if completed successfully, grants the applicant a marketing authorization across all Member States (Scholz, 2015). Once EMA has approved a novel therapeutic intervention, decisions on its real-world use will be made on the Member State level based on country-specific criteria that determine its price setting, reimbursement conditions, and clinical indications.

In recent years however, this paradigm has faced criticism from authors in the field (Mullins et al., 2014; Ioannidis, 2016; Lacombe et al., 2019b; Wieseler et al., 2019) for being too drug-centered and/or for not sufficiently focusing on the patients who will eventually receive the treatment in real-world clinical practice. The existing clinical development framework allows the industry to primarily pursue regulatory approval of their products without taking into account the real needs of patients and society (Lacombe et al., 2019a). In oncology, the growing importance of the precision medicine model, which strives to provide the right patient with the right treatment at the right time through characterization of an individual’s genotypes and phenotypes (European Council, 2015; Salgado et al., 2017), along with its associated costs and limitations (Tannock and Hickman, 2016), has further highlighted the need for more patient-centered drug development (Lacombe et al., 2019c). Nevertheless, clinical cancer research is still too drug-focused at present, as illustrated by the predominance of trials that feature badly chosen comparators which do not reflect the best available therapeutic alternatives (Tao and Prasad, 2018), surrogate endpoints which may not necessarily translate into clinical benefit (e.g. progression-free survival or response rate) (Svensson et al., 2013; Prasad et al., 2015; Kim and Prasad, 2016; Chen et al., 2019), and strictly homogeneous samples of participants representing just 2–4% of the overall targeted population, thereby generating results with a poor external validity (Kennedy-Martin et al., 2015). As such, these studies are not primarily designed to inform clinical practice and do not answer to patients’ needs (Lacombe et al., 2019a), despite serving as the basis for EMA’s decisions to authorize new therapies.

For instance, in a recent retrospective cohort study (Davis et al., 2017), it was concluded that 39 out of the 68 anticancer drugs approved by EMA between 2009 and 2013 entered the market based solely on improvements in surrogate outcomes and without having been shown to increase the overall survival or quality of life of patients, which are outcome measures that can be considered to be truly patient-centered (Kempf et al., 2017). At a minimum of 3.3 years after their registration, there were either still no data available indicating that they prolonged or improved patients’ lives, or the observed gains were more often than not determined to be clinically insignificant. Similar findings were seen in the United States (Kim and Prasad, 2015), where at a median of 4.4 years after their approval, 57% of the antitumor agents registered by the Food and Drug Administration (FDA) between 2008 and 2012 had no or unknown effects on overall survival. An extensive analysis of the FDA’s oncological medicine approvals between 2006 and 2015 affirmed these observations (Rodríguez et al., 2019). Furthermore, clinical trials providing the evidence needed to underpin the regulatory approval process (hereinafter referred to as “registrational trials”) can be prone to bias, which often remains inadequately reported (Naci et al., 2019). Moreover, the accelerated and conditional marketing authorization mechanisms launched by FDA and EMA respectively have further solidified the use of surrogate endpoints in clinical trials (Fleming, 2005; Downing et al., 2014; Hoekman et al., 2015). While these schemes allow promising new therapies addressing unmet medical needs to enter the market faster, the antineoplastic drugs following such expedited approval pathways are rarely shown to increase patients’ overall survival or quality of life in subsequent confirmatory trials (Gyawali et al., 2019; Schuster Bruce et al., 2019). These results suggest that the regulatory approval procedure does not sufficiently filter out medicines that are of limited value to patients and their healthcare providers (Prasad, 2017), which in turn contributes toward the issue of medical reversal, i.e. the costly phenomenon where new, more rigorously designed studies disprove the clinical utility of medical interventions that have been adopted into the healthcare system (Prasad and Cifu, 2011). Additionally, regulators show little interest in rescinding the marketing authorization of cancer therapies that may be ineffective in spite of their exorbitant prices (Rupp and Zuckerman, 2017). In short, it can be stated that the real-life patient is not at the core of the current registrational trials and procedures (Lacombe et al., 2019a).

Although the drug-driven approach to bring new treatments to the market should ideally be balanced with a more patient-focused strategy, the former tends to dominate treatment development in oncology. As a result, many important aspects relating to the use of novel antitumor therapies in real-world settings are neglected throughout the process (Kempf et al., 2017; Lacombe et al., 2017; Lacombe et al., 2019b), as displayed in **Table 1**. For now, such clinically important and patient-centered questions are being addressed in a non-systematic and voluntary manner in the post-approval stage by non-commercial entities (Kempf et al., 2017), including academic research teams and not-for-profit organizations such as the European Organisation for Research and Treatment of Cancer (EORTC), which conducts independent international clinical trials in the field of oncology. However, given the uncertain nature of the funding available for studies performed outside of commercial interests, a more systematic approach is needed (Kempf et al., 2017). The industry has no incentive to invest in this type of research as its results can negatively impact the profitability of their products (Lacombe et al., 2019a), for example when it establishes a shorter overall treatment duration or a lower optimal dose.

**Table 1:** Overview of research concepts and questions that remain underrepresented in clinical cancer research today (Kempf et al., 2017; Lacombe et al., 2017; Lacombe et al., 2019b). The conclusions which may emerge from studies that explore such topics are highly relevant for clinical practice and are shown for a given treatment A.

Research concept	Research question	Possible conclusions
Combination	How and to which extent should the new therapeutic intervention be combined with other existing treatments?	Treatment A should or should not be combined with treatment B for optimal effectiveness
Sequence	In which sequence does the new therapeutic intervention have to be applied when combined with additional therapies?	Treatment A is best given before, after or at the same time as treatment B
Comparison	How well does the new therapeutic intervention perform compared to alternative treatments?	Treatment A is better or worse than or noninferior to standard-of-care treatment B
Performance in real-world patients	How will the new therapeutic intervention perform in patient populations that were excluded from clinical trials?	Specific patient subpopulations may experience better, worse, or similar outcomes when given treatment A compared to the sample of participants included in the clinical studies
Treatment duration	How long does the new therapeutic intervention have to be applied to achieve the desired effects?	Treatment A should be administered for as long the patient lives or may be discontinued after a certain amount of time with no effect on disease outcomes
Dosing	What is the lowest dose at which the new therapeutic intervention can be given without negatively impacting treatment outcomes?	The dosage of treatment A should remain unchanged or may be lowered with no effect on therapeutic outcomes
Long-term outcomes	How do the efficacy, effectiveness and safety of the new therapeutic intervention evolve over a longer period of time?	The efficacy, effectiveness, and/or safety of treatment A may remain stable or decrease over time
Patient-relevant outcomes	How does the new therapeutic intervention perform in terms of patient-relevant outcome measures?	Treatment A may or may not significantly improve patients' perceived health status, quality of life, or overall survival

It is clear that there exists a research gap between the development of anticancer medicines and their use in real-life circumstances (Kempf et al., 2017), resulting in the emergence of two disconnected stages. The first stage is situated in the pre-approval setting and encompasses most of the clinical studies performed today. The research carried out in this stage focuses on characterizing the safety, quality, and efficacy of novel medicinal compounds in highly selected participants and its ultimate goal is to obtain a marketing authorization from the appropriate regulatory authority (Kempf et al., 2017). The second stage, at present comprising a minority of trials conducted, takes place in the post-approval environment and involves the setup of studies that tackle questions intended to accommodate patients’ and clinicians’ needs in real-world clinical practice, such as the ones listed in **Table 1**. Since different actors coordinate the two stages, with the industry taking the lead in the first stage and academia and non-commercial partners playing a more prominent role in the second, the studies organized in the second stage, if done at all, are usually not planned before the drug enters the market, marking a discontinuity that can undermine the optimal implementation of research findings in clinical practice guidelines (Kempf et al., 2017). This situation is not only detrimental to patients, whose true needs are left unaddressed, but also to society as a whole, because it presents a major financial burden to healthcare systems which face growing uncertainty as to the real-life effects and benefits of new health technologies when deciding whether or not to reimburse them, potentially leading to the coverage of less cost-effective treatment options (Lacombe et al., 2019a).

This situation has led to calls for a transition toward a patient-centered paradigm that puts a strong emphasis on applied clinical research (Kempf et al., 2017; Lieu and Platt, 2017; Lacombe et al., 2019a; Lacombe et al., 2019b) Applied clinical research, which has also been described as treatment optimization (EORTC, 2019; Lacombe et al., 2019c), can be defined as optimizing the way treatments are utilized in real-world conditions through the conduct of studies set up to provide an answer to one or multiple of the research questions shown in **Table 1** (Kempf et al., 2017; Lacombe et al., 2019a). It is not intended to replace the current drug development trials; instead, it seeks to deliver results complementing those of the registrational studies as part of a streamlined process that bridges the gap between the first and second stage research (Kempf et al., 2017; Lacombe et al., 2019b). While no definitive methodological framework has yet been formulated for such treatment optimization research, it is likely that prospective designs capable of producing robust level I evidence will be required to reduce uncertainty and improve the acceptability of study outcomes (Lacombe et al., 2019a; Lacombe et al., 2019c). Although population-level observational studies such as those based on cancer registries could provide useful data on long-term outcomes of treatments (Brewster et al., 2005), doubts have been raised as to whether these real-world data collection schemes can replace the conduct of randomized controlled trials for the evaluation of therapeutic effects (Giordano et al., 2008; McGale et al., 2016). Nevertheless, an integrated treatment optimization approach incorporating both interventional and observational research could be a way forward (Kempf et al., 2017; Lacombe et al., 2019a).

However, a number of important questions remain. For instance, it is not yet clear how applied clinical research should be financed: is it the responsibility of the manufacturer to provide the appropriate funding, or should it be fully or partially covered by our healthcare systems? Another aspect that demands further attention relates to the timing of treatment optimization studies: can they run in parallel with the classical registrational trials, or should they take place only after the marketing authorization has been granted? Furthermore, the extent to which regulatory agencies or payers should impose applied clinical research and consider its outcomes during their (re-)evaluation of product dossiers requires clarification. Given the multi-stakeholder nature of the environment in which drug development takes place and the oftentimes conflicting goals and motivations that may arise in such a setting (Kempf et al., 2017), input on these remaining questions should be gathered from all actors involved in the process and considered thoroughly before launching any initiatives to implement treatment optimization into the existing clinical research paradigm. In this study, we set out to investigate the views of key drug development stakeholders concerning the subject of treatment optimization studies so as to probe their acceptability among relevant experts as well as to provide a first overview of their optimal organizational and design features according to them.

## Methods

Semi-structured interviews were performed with experts belonging to one of six different groups of stakeholders in the drug development process, namely 1) academic clinicians, 2) patient organization representatives, 3) regulator representatives, 4) health technology assessment (HTA) agency representatives, 5) payer representatives, and 6) representatives of the pharmaceutical industry. Participants were asked about the current situation in drug development as well as the optimal features of treatment optimization studies. The interview questions were derived from relevant literature on the topics of treatment optimization and the aforementioned evidence gap (Mullins et al., 2014; Ioannidis, 2016; Kempf et al., 2017; Lieu and Platt, 2017; EORTC, 2019; Lacombe et al., 2019b; Lacombe et al., 2019a). The full questions are listed in the **Supplementary Material**. All interviewees were asked the same questions in the same order to allow for inter-group comparisons and to minimize the risk of order effects bias. However, some questions had to be adapted to accommodate the individual stakeholder groups. Moreover, depending on the answers that were provided by the interviewees, some additional questions may have been asked with the intent of further clarifying their standpoints.

Participants were recruited through a combination of purposive and snowball sampling. Prior to recruitment, a list of inclusion criteria (**Table 2**) was composed to facilitate the identification of suitable interviewees. Since the study aimed to examine the perspectives of European stakeholders, delegates of institutions and organizations that are active on a European level were included wherever possible. Selected experts were invited to participate by e-mail and received the interview questions in advance. This was done to increase their understanding of the recently introduced (EORTC, 2019; Lacombe et al., 2019c) concept of treatment optimization and to improve the overall flow of the interview sessions.

**Table 2:** List of inclusion criteria used to recruit representatives of each stakeholder group included in the study.

Stakeholder group	Inclusion criteria for recruitment of representatives
Pharmaceutical industry	Is in a senior or upper management position Has been a member of a clinical drug development team before or has expertise in real-world evidence Speaks fluent English Works or has worked in a Member State of the European Union
Patient organizations	Has experience working as a professional patient representative Has knowledge of clinical drug development Speaks fluent English Works or has worked in a Member State of the European Union
HTA agencies	Is in a position of authority at an HTA agency Is actively involved in decision-making Speaks fluent English Works or has worked in a Member State of the European Union
Regulators	Is in a position of authority at a national medicines regulator or at the European Medicines Agency Is actively involved in decision-making Speaks fluent English Works or has worked in a Member State of the European Union
Payers	Is in a position of authority at a government agency responsible for drug reimbursement decisions or at the expert body advising said agency Is actively involved in decision-making Speaks fluent English Works or has worked in a Member State of the European Union
Academic clinicians	Has been involved in phase III trials as a principal investigator Has a senior position at a university hospital Is a member of a scientific society such as the European Society for Medical Oncology (ESMO) Speaks fluent English Works or has worked in a Member State of the European Union

**Figure 1:**
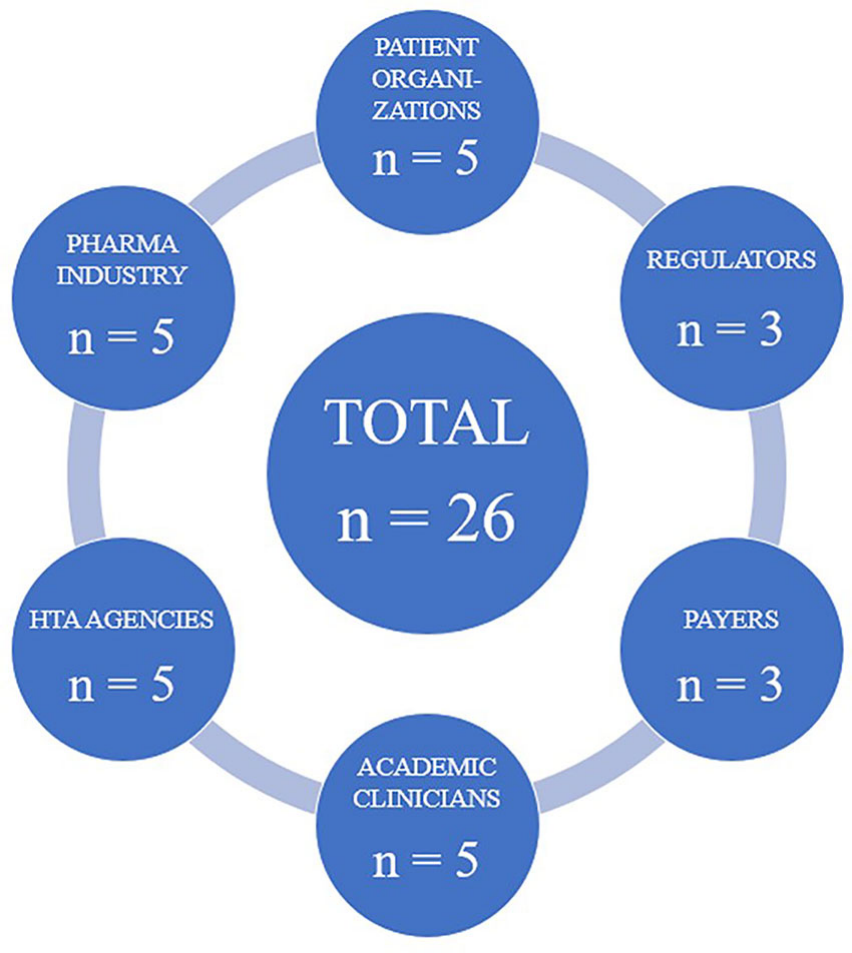
Visual representation of the stakeholder groups included in the study and the number of participants recruited for each group.

In total, 26 interviews were carried out between December 2018 and May 2019. All interviewees were working in EU Member States at the time of their interviews. The countries included were Austria, Belgium, France, Germany, Italy, the Netherlands, Poland, Spain, Sweden, and the United Kingdom. As illustrated in **Figure 1**, an even distribution of participants across the targeted stakeholder groups was achieved. Although the majority of interviewees had expertise in oncology, there were also experts from other medical fields who took part in the study, including rheumatology, hematology, and pneumology. The industry representatives were affiliated with pharmaceutical industry associations or large multinational drug companies. The participants representing patient organizations were all professionally employed by European-wide advocacy groups for either rare or common diseases. The countries from which the HTA agency representatives were recruited were Austria, Belgium, the Netherlands, Sweden, and the United Kingdom, while the participating payers voiced the Austrian and Belgian perspectives. For the regulator representatives, experts from both EMA and national regulatory authorities (specifically, the Medicines and Healthcare Products Regulatory Agency from the United Kingdom and the Spanish Agency of Medicines and Medical Devices) were interviewed.

The interviews were conducted *via* Skype^®^ and audio-recorded digitally. To minimize bias as a result of interviewer variance, one researcher was responsible for performing all interviews. The recordings were pseudonymized and subsequently transcribed *ad verbatim* by a third-party company. The transcripts were analyzed based on the framework method (Gale et al., 2013; Spencer et al., 2014) using the NVivo^®^ software.

## Results

### Current Situation in Drug Development

#### Focus of Current Drug Development Framework

Most interviewees (22 out of 26) agreed with the notion that drug development research is not sufficiently patient-centered. They believed that patients are not being adequately involved at the design stage of clinical studies, when the research questions are defined and the protocols set up. Furthermore, at the time of approval, very little knowledge is available on the tangible benefits of a new therapeutic intervention for the patient, according to these participants.

“*And for the moment, of course, the way the research is organized is to get marketing authorization as fast as possible [ … ] but the more patient-oriented clinical relevancy, added therapeutic value, or notions like quality of life evidence are I think rarely taken into account and into consideration when designing the pivotal studies.*”

Payer representative 2

“*And patient involvement today is very strongly still window dressing, where patients are involved by the time when it’s about informed consent documents, thank you letters, or if there are recruitment problems [ … ]*”

Patient organization representative 5

However, there was no agreement on whether drug development research is also too drug-focused. For instance, many participants (10 out of 26) highlighted the necessity of the present drug-centered attitude to ensure the treatment is safe, efficacious, and of sufficient quality. Nevertheless, they added that it should be balanced with a more patient-focused strategy which addresses patients’ real needs, stressing that the two types of approaches are not incompatible but even complementary with one another.

“*I agree with this notion, but if we want to be successful, I think we need to work together with industry and to add on top of the current drug development research the more patient-centered applied clinical research. So it’s not ‘or’, it’s ‘and’.*”

Academic clinician 1

“*I mean, it’s fine to develop the product on the basis of a development which is brackets ‘drug-centered’ [ … ] I think the concern is after the authorization, whereas you should have clinical trials which are developed and which should basically inform the clinical practice, these trials do not take place and are not part of the post-authorization commitments that the companies have to fulfill.*”

HTA agency representative 3

Several interviewees (8 out of 26) thought that the system is, for a variety of reasons, undergoing a major shift toward implementing a paradigm that puts the patient at the center of the development process.

“*So in the industry, we are drug-centered as you can imagine because our ultimate goal is to deliver therapeutic solutions which will help [interviewee’s employer]’s shareholders making benefit. [ … ] If we are too drug-centered, we would have some issues in barely selling our drug stock. We are more and more patient-centered because there are more and more pressures to be patient-centered.*”

Industry representative 1

“*So if you look at the bulk of clinical trials, then yes, they are too much molecule-centered [ … ] But I can also see some shift in this paradigm. So I can see that there is improvement in this regard and it’s a recent development; so it’s new. But we should still acknowledge that it is happening.*”

Patient organization representative 4

“*This process becomes more patient-centric and would become more patient-centric over time because of science, personalization and technology that allows better engagement and involvement of patients in medicines development.*”

Industry representative 3

Participants who disagreed with the notion that drug development research is too drug-centered and insufficiently patient-centered (2 out of 26) either believed it is already strongly patient-driven today or were of the opinion that the terms “drug-centered” and “patient-centered” were too simplistic to describe the current system and that the perceived dichotomy between the two is false.

“*In all our studies, we have significant effort to get the patient perspective and the patient experience documented. [ … ] So, I’d say as an industry and in [interviewee’s employer], we’re actually very patient-centric in our drug development process.*”

Industry representative 4

“*If by drug-centered you mean that companies conduct drug development which aims to establish quality, safety, efficacy, and positive benefit risk to meet the legal requirement for marketing authorization, then yes, the development is drug-centered. But I find both terms are unnecessarily simplistic in the sense that a drug which has shown those characteristics and that is put on the market can benefit patients.*”

Regulator representative 1

#### Impact of Current Drug Development Approach

The interviewees who believed that the current drug development paradigm is too drug-centered were convinced that the present approach severely complicates the decision-making of HTA bodies, payers, and clinicians. As reimbursement assessments are based on the available results from clinical trials, a costly new drug may be reimbursed for a population of patients that is either as narrow as or broader than the sample of trial participants. This could limit patients’ access to the treatment or introduce uncertainties with respect to the clinical utility of the therapeutic intervention, respectively. In the latter case, a lower price may be negotiated or a managed entry agreement can be set up to mediate further data collection. An overly strong focus on the approval and commercialization of the drug may therefore result in missed opportunities for patients and impact the way physicians operate in clinical practice, since their treatment decisions are influenced by evidence-based guidelines and financial considerations.

“*Basically, you don’t have any utility data, you only have very rough efficacy data. [ … ] That might be enough to determine the benefit risk ratio, but it’s not enough for determining the value of the drug. So we really don’t know what we’re gonna pay for the drugs right now and in the future, if this trend goes on.*”

HTA agency representative 4

“*I think looking for the commercially viable treatment has crowded out some quite good therapeutic ideas. [ … ] It also means that combination studies don’t happen, because drug A from pharma A, and drug B from pharma B, neither of them wants to work with the other pharma company. Similarly, we don’t get comparison studies, drug A versus drug B, for the same reasons. And, you know, this drive to be first to market, [ … ] that first to market concept is very important to pharmaceutical companies. [ … ] And it distorts the market because some things come to market which, frankly, at the end of the day, might best not be bothering.*”

Patient organization representative 2

The regulator representatives were either of the opinion that the evidence generated through the application of the existing clinical research framework is of sufficient value to underpin regulatory decision-making, or argued that efforts to increase patient involvement in clinical trial design, however valuable they may be, make the assessment of marketing authorization applications more challenging.

“*I think it’s more challenging when you bring the patient voice alongside the regulator voice and other voices because you obviously have another stakeholder to consider and weigh up. [ … ] So, you know, how do you take on their views in a way that’s robust, but is also representative of the patient group that you want to consult? [ … ] Patient views provide an essential, I think, addition to the views of either regulatory experts or healthcare professionals, but I think it doesn’t make it necessarily easier to make the decisions.*”

Regulator representative 2

“*Our job, you see, is just to evaluate a new drug, no? And of course, we have to involve patients in the clinical trial designs. We have to involve patients in the questions that we want to get from the clinical trials. But in the end, this is a clinical trial that is trying to elucidate the effect of a drug.*”

Regulator representative 3

The industry representatives, all of whom thought that the industry is increasingly engaging in patient-centered clinical research, asserted that initiatives to involve patients in the drug development process are being undertaken by pharmaceutical companies as a way to convince regulators, HTA bodies, and payers of the added value of their products for the patients. This in turn should translate into reimbursement conditions that are favorable to the manufacturers. Furthermore, such efforts fall in line with recent trends to deliver more personalized treatment strategies.

“*The value that we create and that would be recognized by the payers in the future would also depend on the impact on the patient and society at large and not only the performance of a molecule in an assay. [ … ] So yes, in the end it is recognition of the value and then willingness to reimburse and also defining new types of reimbursement models and managed entry agreements in the future.*”

Industry representative 3

“*I think it’s a sense of making sure our research really delivers what patients need and what’s really important to them and recognizing that they are the ultimate end users of the medicines. And it’s about improving patients’ health. I think also we’re seeing more demands from our payer, HTA environments and our regulatory environments.*”

Industry representative 4

#### Real-World Evidence

The majority of interviewees (18 out of 26) agreed with the assertion that there is insufficient real-world evidence (RWE) underlying the use of many drugs on the market today (note that RWE is defined here as the evidence establishing the effectiveness and applicability of a therapeutic intervention in real-life clinical practice). When asked to explain the sparse availability of such evidence, these participants listed a number of different reasons.

First of all, the collection of real-world data (RWD) from which RWE can be derived is complex, often requiring the application of advanced data acquisition systems. This makes it difficult and costly for pharmaceutical companies to routinely gather such information. Privacy and data protection laws present additional hurdles to performing real-world effectiveness studies, especially in Europe, where the recently introduced General Data Protection Regulation could undermine data access and sharing according to two interviewees. Moreover, there are significant differences between individual countries in what exactly constitutes clinical practice, which further complicates the gathering of RWD.

Secondly, the European regulatory landscape is fragmented, with the marketing authorization being granted at the European level and the decision to reimburse the drug taking place at the national level. Both processes involve the review of available data but focus on different aspects. The lack of systematic communication between the regulators on the one hand and the payers and HTA agencies on the other leads to the industry prioritizing the requirements for regulatory approval by EMA, which only examines information on the quality, safety, and efficacy of the drug and leaves its effectiveness out of consideration. For their subsequent assessments, HTA agencies and payers therefore often cannot rely on the availability of RWE showing that manufacturers’ products are actually effective in clinical practice.

Thirdly, even if RWD are available, they are usually deemed of inadequate quality to inform decision-makers. One interviewee even used the term “dirty data” when describing the limited utility of the information derived from most RWE studies today. The lack of therapeutic standardization in real-life environments is a major contributing factor to this issue: if there is no standardized way to prescribe a new treatment, it is not immediately clear whether an apparent lack of effect seen in clinical practice is caused by the drug not being effective, or the result of it not being applied in the optimal way. Furthermore, if there is no comparison between the investigational therapy and a particular control treatment and/or no randomization, the data obtained will be considered less reliable.

Lastly, the manufacturer is usually not obligated to conduct studies designed to assess the effectiveness of new treatments, so there is no pressure on the industry to actually undertake them. As there is no overarching regulatory framework in place, no one is responsible for collecting RWD at present. There are also no real incentives for any of the actors in the drug development process to take the lead and launch spontaneous efforts to gather such data. For instance, most clinicians would likely not be prepared to perform any supplementary administrative work without being paid an honorarium for doing so, and the industry might refrain from setting up any initiatives in this area because they could uncover additional drug complications that were not detected during the preceding clinical trials. Companies are eager to get their products on the market as soon as possible, supported by regulatory mechanisms to accelerate the approval process and demands of patients and patient organizations for faster access to innovative treatments, while not always sufficiently delivering on their commitments to collect fit-for-purpose RWD in the post-authorization real-life environment.

Most of the interviewees who did not feel it was accurate to say that there is a lack of RWE for the use of many therapies available on the market today (6 out of 8) argued that large quantities of RWD are already being captured and stored by pharmaceutical companies, as illustrated by some of the projects launched with support from the industry in the context of the Innovative Medicines Initiative. Nevertheless, these participants underscored that there was a strong need for gathering such data in a more well-structured, standardized, and transparent manner so that regulatory authorities would be increasingly willing to take this type of evidence into account for their assessments.

Additionally, several interviewees (6 out of 26) emphasized that the concept of RWE lacks a standardized definition, which influenced their understanding of the question and thus also their answers.

“*The concept of real-world evidence is not very well-defined. I mean, it’s not consensual. [ … ] I have to say, I’m not very keen on this word. I would say that there is more a lack of pragmatic evidence or clinical effectiveness evidence on the use of many products on the market today.*”

HTA agency representative 3

### Optimal Features of Treatment Optimization Studies

#### Conduct of Treatment Optimization Studies

When asked which stakeholder(s) should be responsible for the conduct of treatment optimization studies, the interviewees gave divergent answers. Nevertheless, two main options emerged from their responses.

The first option consisted of having treatment optimization studies be performed by academic groups and not-for-profit organizations in the form of independent research institutions, not only because these actors have no ulterior commercial motives, but also due to their prior experience and expertise in this area. Moreover, compared to the industry, they are more pragmatically inclined and more aware of what exactly constitutes real-world clinical practice. However, while manufacturers might have no incentive to engage in applied clinical research, they could still be involved in this process, for example by supplying the coordinating investigators with the study drug at a reduced price or even free of charge.

“*To me, it should be academia and also non-profit organizations, with the support of pharma industry. Support should be both drug supply or some form of discount on drug costs and even budget support. [ … ] The data should be owned by academia, non-profit organization of course, and it should be scrutinized and analyzed independently from a commercial interest.*”

Academic clinician 2

In the second option, treatment optimization studies would be undertaken by consortia or collaborative groups comprised of all relevant stakeholders. Proponents of this scenario were convinced that these trials should not be carried out by any single particular actor, as their results are useful for everyone involved in medicines development.

“*I think that consortia of all of these stakeholders should be involved, that’s what I think. And it doesn’t matter who leads, the bottom line is that everybody should be part of the research teams, all the stakeholders, because that will speed up the approval process, that will streamline the outcomes. [ … ] I mean, it’s easier to do treatment optimization if everybody has the same understanding of what is the optimum.*”

Patient organization representative 4

#### Funding of Treatment Optimization Studies

Most interviewees (16 out of 26) saw a combination of public and private funding as either a viable or the most viable mechanism for financing treatment optimization research. One of the main arguments given in support of this position was that the conduct of these studies should be independent from the commercial pressure of the pharmaceutical industry in order to prevent bias, but that at the same time, academia or not-for-profit organizations do not have the means to fund them fully on a sufficiently large scale. The industry can then contribute by supplying their drugs at a discount or even for free. Another reason why many interviewees preferred joint funding partnerships was that both the public and commercial sectors would potentially stand to benefit from the conclusions of treatment optimization studies. Healthcare systems could realize major savings and improve patient outcomes, while pharmaceutical companies could increase their revenues and negotiate more favorable reimbursement conditions in such a scenario. Furthermore, a good mix between public and private sponsorship of applied clinical research ensures a balanced protocol and prevents the introduction of bias from either side.

*“For me, it should definitely be a combination of the two. [ … ] If these studies are necessary, it’s also because the drug was initially not properly characterized, so you cannot expect that the company continues to make huge benefits and that on the other side a non-commercial, a non-profit organization or the authority or the academia do the study to fill this gap on their own funds.*”

Payer representative 1

An important point that some interviewees (4 out of 26) brought up was that no matter how treatment optimization is going to be financed, choices will have to be made regarding which topics and medicines to focus on as there will not be infinite resources to spend on these studies. Therapies can be optimized endlessly and there are countless amounts of treatment combinations that can be investigated in oncology. The therapeutic areas and products that are affected the most by the current lack of applied clinical research need to be identified and given priority.

#### Timing of Treatment Optimization Studies

There was no clear consensus among the people interviewed concerning the optimal timing of treatment optimization studies. Many participants (15 out of 26) were convinced that they could already be initiated before the therapy in question has been approved by the regulatory authorities, while others (11 out of 26) considered the post-authorization conduct of these trials to be the most realistic option.

“*If you have a joint HTA-regulatory system, then you could have them already during phase three and not lose time, because if you have to wait until the product is on the market and then you have to set up your treatment optimization studies, you lose five or ten years. So, the earlier you start with them and then the earlier you think about it, before the phase three preferably, the better.*”

HTA agency representative 1

“*If the drug is not yet marketed, is not yet approved by EMA, it’s extremely difficult to perform the study without the drug company. So the drug company will be not only the provider, supplier of the drug, but also then design the study, perform the trial, and will be in the driver’s seat. And then, of course, you have an increased risk of bias.*”

Academic clinician 1

Nevertheless, some of the interviewees (3 out of 11) who saw treatment optimization studies taking place exclusively in the post-approval stage of the drug development process emphasized that the questions to address and the type of information to collect should preferably be defined as early as possible, for example during phase three, so that applied clinical research can start immediately after the therapy has received marketing authorization.

#### Design Features of Treatment Optimization Studies

The participants mentioned a number of design elements that according to them should be incorporated into treatment optimization studies so that their results may be as relevant as possible for clinical practice.

##### Patient Selection

With respect to the recruitment, most interviewees (20 out of 26) asserted that fewer exclusion criteria should be applied, and that the effects of the drug should be examined in more diverse subpopulations, e.g. patients with comorbidities, elderly patients, smokers, patients taking multiple additional medications, cancer patients with a poor performance status, etc. Moreover, trial subjects should not solely be recruited by academic clinicians in university hospitals, but also by primary care physicians or specialists working in smaller or private hospitals. A more pragmatic and less stringent selection procedure will generate a sample of participants that is reflective of the true patient population. The focus should lie on the real patient, rather than the ideal one. Current recruitment strategies, while useful for demonstrating the efficacy of therapeutic interventions, can slow down the development process and have made pharmaceutical companies look to less developed countries in order to find patients who satisfy all the inclusion criteria and who do not have access to effective alternative treatments. However, this raises questions about the applicability of the eventual findings to patient populations in Western countries. Nevertheless, these conventional methods for selecting participants should not be abandoned, but simply applied in the right context, namely that of the classical registrational trials. Treatment optimization studies should adopt a broader perspective.

##### Randomization

Many participants (13 out of 26) believed that randomization would still be necessary in treatment optimization studies to reduce bias. Without randomization, the results of treatment optimization studies will likely not be considered robust enough by regulatory agencies, HTA bodies, payers, and clinicians to inform their decision-making, according to them. However, they also acknowledged that it is not always feasible to randomize patients to parallel treatment arms (e.g. in rare diseases). Other interviewees (11 out of 26) saw randomization as a barrier to simulating real-world conditions, since patients and doctors outside of trial settings can actively choose which treatments they will undergo or administer. As long as its omission does not undermine the statistical analysis of the trial data, randomization would not be needed, one participant remarked. The two remaining interviewees refrained from commenting on this matter.

##### Blinding

Similarly, there was disagreement among the interviewees on whether blinding would be required in treatment optimization research. Several participants (9 out of 26) thought that the act of blinding the trial subjects and investigators to the intervention they were allocated to receive or administer would be necessary to increase the validity of the results. Conversely, others (13 out of 26) believed that it would ultimately diminish the value of the conclusions, as patients and physicians in real-life clinical practice are actually aware of which treatment they are receiving or prescribing. Perceptions they have about the medicine can influence its effectiveness but are ignored in blinded studies, which is why an open-label setting would be preferred by these interviewees. In addition, blinding is not always feasible, and patients today may be technologically adept enough to figure out which treatment arm they were assigned to by looking up information on the internet and communicating with each other *via* social media. Four interviewees did not wish to address the aspect of blinding altogether.

##### Comparator Treatment

The majority (12 out of 16) of the interviewees who shared their views on the nature of the therapeutic intervention with which the investigational drug should be compared in treatment optimization studies were of the opinion that active comparators should be used, and that they should constitute the standard of care or the best available alternative treatments. Since what is considered standard of care varies widely between different regions, the comparator may differ from country to country as well. An important caveat here is that this could introduce a bias in the sense that new treatments will typically be adopted by university hospitals first, so if the new drug is then compared with an existing, widely applied therapy, any differences in effect which are observed could also be attributed to the disparity in settings (academic versus general hospital). The comparator should not be chosen based on the effect size required to match or surpass its performance in a clinical trial, as is often the case today. The comparison in question is only valid if the way the control treatment is applied reflects how it is utilized in real-world clinical practice.

##### Outcome Measures and Endpoints

According to most participants (18 out of 26), the outcomes of treatment optimization studies must be relevant for patients and should be able to objectively express the drug’s effects in real-world conditions. Some of the examples of useful outcomes quoted by the interviewees were quality of life, overall survival, time to treatment failure, treatment duration, long-term effectiveness and toxicity, and patient-reported outcomes. The use of surrogate endpoints should be avoided in applied clinical research, many participants (11 out of 26) believed.

##### Reporting of Results

Concerning the reporting of the results, a recurring sentiment among the participants who addressed this facet (9 out of 10) was that all data should be published, regardless of whether they reflect well upon the investigational drug. New journals could be established for this purpose, since the current high-impact ones favor classical phase II or III studies with positive results. Transparency is key because the findings of treatment optimization research need to inform regulatory, HTA, payer, and clinical decision-making.

### Geographical Setting of Treatment Optimization Studies

Most participants (15 out of 26) did not express a preference for a particular setting, stating that this is something that should be decided on a case-by-case basis, since it depends on the type of disease and intervention that are being investigated in the study. Rare disorders and orphan drugs would for instance benefit from an international approach, whereas infectious diseases are often restricted to specific geographic areas, making a locally or nationally conducted trial the most obvious choice. Additionally, there are merits as well as limitations associated with each possible setting, which underscores the need of having prior knowledge of the context in which a treatment optimization study will be organized before singling out a specific setting as the optimal one.

However, some interviewees (4 out of 26) explicitly favored an international setting, arguing that it would facilitate patient recruitment, increase the efficiency of the research process (one protocol for multiple countries), and improve the acceptability of the findings among regulators. Others (7 out of 26) preferred that such trials would be performed nationally, justifying their choice with the following arguments: standards of care can vary widely between different countries, making it difficult to design studies with overarching objectives relevant for all included nations. Even on just the European level, the treatment landscape for many diseases can be very fragmented. In addition, the effectiveness of drugs can be influenced by ethnicity and genetic diversity, further complicating international treatment optimization efforts. Hence, since it is important for clinicians to have access to information that is applicable to the patients they see in daily clinical practice, the conduct of applied clinical research would have to be limited to environments in which there is a certain degree of homogeneity in the availability of different therapeutic options as well as the genetic profile of potential participants. Furthermore, HTA agencies and reimbursement authorities operate on a national level and therefore place great value on country-level data. Moreover, an international setting would necessitate intense collaboration between countries with possibly very dissimilar healthcare systems and economies, and coming to agreements regarding funding and data ownership could pose a major challenge.

Nevertheless, the majority (5 out of 7) of the participants favoring a national setting indicated that international coordination and oversight of nationally organized treatment optimization studies still remains possible. Protocols could be exchanged and data shared between countries to ensure learnings are implemented and results can be compared. Additionally, efforts to introduce more standardization into the conduct of applied clinical research are best undertaken through the creation of international guidelines.

“*With regard to more standardization and definition and characterization of the data we want to collect, to be relevant, this should be decided at an international level.*”

Payer representative 1

### Inter-Stakeholder Collaboration in the Design of Treatment Optimization Studies

The experts interviewed strongly advocated an inter-stakeholder collaborative approach for the design of treatment optimization studies.

#### Participating Stakeholder Groups

All participants believed that the members of their stakeholder group would be interested in contributing to the design of treatment optimization studies. When the interviewees were subsequently asked who else should be present at the table during the planning stages of such trials, the following collective of actors involved in the development of new medicines emerged from their answers: 1) patients and/or patient organizations; 2) physicians and clinicians (from both academic and community hospitals); 3) academia and independent research organizations; 4) pharmaceutical industry; 5) regulators; 6) HTA agencies; 7) payers.

Some interviewees mentioned that European policymakers (i.e. the European Commission; 3 out of 26), national lawmakers (i.e. the Ministries of Health of the various Member States; 2 out of 26), contract research organizations (if their services are needed; 2 out of 26), and scientific societies (3 out of 26) like the European Society for Medical Oncology (ESMO) could also be invited to take part. Other participants believed that additional healthcare professionals such as nurses (2 out of 26) and pharmacists (1 out of 26) should be included as well, the former due to their day-to-day care of the patient and their awareness of what exactly comprises real-world clinical practice, the latter because of their knowledge of the treatment’s potential for interactions with any concomitant therapies.

#### Contribution of Stakeholder Groups

The academic clinicians highlighted their knowledge of the underlying pathophysiology of the disease as well as the present treatment landscape. Their awareness of the unmet medical needs would help identify the therapeutic areas in which treatment optimization is needed the most. Additionally, their direct contact with patients allows them to better understand what can be done to optimize therapies from a patient-centered point of view. Many academic clinicians are also experienced investigators and are familiar with clinical trial design. Furthermore, they could have some suggestions for Research Topics to investigate, including promising combinations, lower dosage strengths, or reduced treatment durations, based on their observations regarding off-label use of registered medicines in real-life clinical practice.

The HTA representatives thought the input of their agencies would reflect the activities they are currently already doing, such as performing systematic literature reviews and making sure that the results of the studies can be interpreted by health economists for the purpose of producing cost-effectiveness assessments. Moreover, these inter-stakeholder dialogues would present them with the opportunity to express which type of data they need for their decision-making and to voice their concerns about the selected outcome measures or comparators that might not be appropriate for predicting clinical utility.

Similarly, the payer representatives saw a multi-stakeholder platform as the ideal environment to provide further clarification on the type of evidence they require for their decision-making. Moreover, they asserted that they would utilize such an opportunity to accentuate potential gaps in the available data and to recommend potential Research Topics that could be addressed in treatment optimization trials. The regulator representatives on the other hand were of the opinion that the regulatory authorities could assist in assessing the methodology of applied clinical research and facilitating the dialogue between academia and the industry.

The patient organization representatives thought that patient organizations could defend the interests of patients, giving them a platform to convey what they expect of treatment optimization studies and to ensure that the conclusions of these trials are of value to them. In addition, they have a much more end-to-end mindset than most other stakeholders, meaning that they will approach the Research Topics backwards and first ask themselves what the real-world application of the product will be. They can also help ensure that the benefit–risk balance of taking part in the trial remains positive by assessing whether the burden imposed on the participants does not outweigh the therapeutic effects they are expected to experience.

The industry representatives underscored the industry’s experience with conducting clinical trials, as demonstrated by the complex systems they have in place and the networks they have built up over the years with clinicians around the world. Pharmaceutical companies also have a wealth of expertise at their disposal, which would facilitate the design and conduct of treatment optimization studies.

#### Hurdles to Stakeholder Participation

Several challenges were cited by the interviewees that could jeopardize their willingness to devote themselves to the coordination of applied clinical research.

The academic clinicians worried that they would be compensated less than for classical randomized controlled trials, given the limited funding that is currently being made available for treatment optimization research. Additionally, the results of pivotal phase II or III trials are typically published in high-impact scientific journals, thereby furthering the academic careers of the clinicians involved. The interviewees did not anticipate the same kind of recognition emerging from their involvement in treatment optimization studies. Both of these aspects would be especially problematic when clinicians have to choose between contributing to industry-sponsored trials, which offer them a multitude of perks in exchange for their time and efforts, or to treatment optimization studies, which likely cannot match those benefits.

The HTA representatives warned that their agencies are already under-resourced and under-staffed for their present responsibilities. Moreover, HTA involvement could introduce a bias, since HTA agencies are probably more inclined to produce favorable assessments for medicines whose development they were actively involved in. A similar argument was brought up by one of the regulator representatives, who did not think the regulator should have a say in how treatment optimization trials are designed out of concern that the risk for conflicts of interest to arise would be too great.

The payer representatives noted that healthcare payers do not have the resources to join discussions surrounding the design of treatment optimization studies every time a new trial is planned. Instead, they would only participate occasionally, mostly when the impact of these studies is expected to be large, such as in therapeutic areas where there is an urgent need for more applied clinical research. If the payers do not derive any concrete gains in terms of their own goals and objectives from their participation in these dialogues, they will halt their support and refuse to attend future meetings.

Although the patient organization representatives showed great enthusiasm about the prospect of being invited to discuss the design of treatment optimization studies, they also openly admitted that most patients and patient organizations are not ready yet to commit themselves to such an advisory role in a meaningful way. It would require a complete overhaul of the current system, with the creation of new methodologies and strategies to find patients and patient advocacy groups and subsequently recruit, train, mentor, involve, and support them. Without the implementation of such changes, it would be too difficult to generate useful results from the incorporation of the patient perspective.

### Regulatory Measures to Support Treatment Optimization

There was unanimous support among the participants for regulatory measures to facilitate and support treatment optimization, although there was no agreement on the optimal size, scale, and nature of these initiatives.

Some interviewees (18 out of 26) favored strong action and wanted to make it mandatory for the industry to undertake applied clinical research. To realize this, three different strategies were proposed.

The first strategy involves making the conduct of treatment optimization studies part of the requirements that manufacturers have to satisfy in order to obtain a marketing authorization for their products. If a company does not provide the requested information or if the results indicate that there is insufficient reason to believe the new medicine will be useful in clinical practice, approval would not be granted.

The second strategy consists of including treatment optimization studies as part of the post-authorization commitments that are imposed on the industry in the context of EMA’s conditional approval procedure. In this scenario, EMA would grant a marketing authorization to the applicant based on the data that was acquired from the standard registrational trials, on the condition that additional evidence derived from applied clinical research is presented within a predetermined timeframe. If this condition is not met or if the findings cast doubt upon the clinical utility of the intervention, the approval would be retracted or adapted (e.g. by narrowing down the patient population that can receive the medicine).

In the third strategy, conditional reimbursement mechanisms would be employed to compel the manufacturers to carry out treatment optimization studies. This means that the national payer would temporarily reimburse the treatment while the manufacturer collects supplementary evidence in the form of applied clinical research data. If the information requested is not provided within a predefined number of years or if the results reflect negatively upon the therapy in question, the reimbursement could be halted or the conditions under which it was negotiated may be altered.

Other participants (8 out of 26) feared that making treatment optimization compulsory would ultimately prolong the time to approval and/or reimbursement, thereby increasing the costs of drug development and preventing timely access of patients to novel therapies. Instead, they expressed their preference for an approach in which applied clinical research would be incentivized rather than mandated, for example by offering companies which undertake treatment optimization studies extended periods of market or data exclusivity. Moreover, according to these interviewees, the regulatory authorities could take measures to promote applied clinical research by coordinating workshops, debates, and discussions on this topic, setting new assessment criteria for trials of this type, and giving early scientific advice to stakeholders involved in their conduct.

### Opportunities and Challenges of Treatment Optimization Studies

The interviewees listed a multitude of different opportunities and challenges which they associated with the conduct of treatment optimization studies. These are summarized in **Table 3**.

**Table 3:** Opportunities and challenges of treatment optimization studies that were named by the interviewees. Note that some points can be considered as both an opportunity and a challenge, depending on which stakeholder's perspective one chooses to adopt (e.g. increased drug prices can be beneficial to the industry but detrimental to patients).

Opportunities	Challenges
Patients would benefit directly from its results, since its objectives and outcomes measures were selected based on their relevance for clinical practice	The financing of such studies poses a challenge, especially since they will likely have to run over a long period of time and include a large number of participants: sponsoring by the industry could allow them to influence the trial setup and increase their drug prices, while public funding could be seen as a double payment and an unacceptable shift of the financial burden linked with developing new drugs toward the public
It could improve HTA and payer decision-making through prevention or identification of inappropriate reimbursement decisions, thereby decreasing costs and increasing healthcare spending efficiency	The industry might not want to be involved if the probability of a favorable outcome for their drugs is too low, given the loss of revenue such initiatives could cause
It could improve clinical decision-making by providing physicians with evidence that a therapy works when it is applied in real-world conditions, as well as with information on how it should be administered to achieve the best results	Clinicians might not be willing to participate due to the additional burden imposed on them, their inexperience with this kind of research and the lack of interest on the part of high-impact scientific journals to publish its results
It could give manufacturers' products a major marketing advantage over those of their competitors, which could translate into higher prices, more favorable reimbursement conditions, and an increased uptake by clinicians	No general framework surrounding the optimal design and methodological features of such studies has been created yet, and the associated uncertainties can only be managed through the use of larger sample sizes, the development of quality standards, and other measures of this kind
It could lead to the registration of additional indications in specific subpopulations and generate new combinations of active substances, resulting in the broader application of drugs in clinical practice	The infrastructure needed to perform these studies in a multi-stakeholder and potentially international manner is currently not yet available and could potentially give rise to conflicts of interest
It would allow us to identify and reward those medicines that have an added clinical value compared to existing alternatives, which could discourage the development of me-too drugs with little additional benefit	It could be complicated by legal issues relating to who is liable if unexpected side effects occur when the therapy is used in ways that have not been previously approved, or to who should be able to request changes to the label if the findings of the study warrant such modifications
It could help fill the evidence gaps that are left by the conventional registrational trials at the end of the clinical development program	Its optimal timing remains unclear: in the pre-approval setting, it could delay marketing authorization and therefore patient access, while in the post-authorization environment, its findings may come too late to influence regulatory or payer decision-making

### Evidence Strength of Treatment Optimization Studies

Most participants (18 out of 26) believed that treatment optimization studies would generate results whose evidence strength would be greater than (9 out of 26) or at least equal to (9 out of 26) that of the findings obtained from conventional registrational trials. However, they also noted that the superiority or parity in evidence strength is only valid if the optimal design features (see above) are integrated into these studies and if they are performed according to rigorous quality standards. The latter condition can be achieved through implementation of data quality checks, routine monitoring of patient information, verification of source data, standardized measuring of clinical parameters, education and training of investigators, and other efforts of a similar nature.

The majority of the eight remaining interviewees (5 out of 8) refrained from directly comparing treatment optimization studies to classical randomized controlled trials. Three of them stated that both are necessary and can contribute to the totality of evidence that will eventually be reviewed or assessed, so it would not make sense to claim one produces more convincing conclusions than the other. The two other participants either did not feel knowledgeable enough to draw a comparison between applied and classical clinical research, or wanted to wait until guidelines have been developed on the methodology of the former. The three interviewees who considered the evidence strength of the conventional registrational trials to be greater than that of treatment optimization studies all noted that they still saw value in optimizing therapies, for instance because it could help elucidate the underlying mechanisms that determine why some patients will and other patients won’t respond well to a particular medical intervention. Furthermore, while they currently deemed the findings of the classical randomized controlled trials to be of a higher evidentiary standard than those of applied clinical research studies, they also acknowledged that their opinion could change in the future, as more advanced methods to collect and analyze RWD and generate RWE become available.

There was a strong consensus among the experts interviewed that the results of treatment optimization studies should have an impact on regulatory, HTA, payer and/or clinical decision-making. Depending on when they thought applied clinical research is best carried out, the participants argued that the assessments performed during the marketing authorization and/or reimbursement application procedures should take into account data derived from such trials, or that a revision of the decision to reimburse the medicine would be warranted if these procedures had already been concluded and the treatment optimization outcomes reflected negatively upon the drug. There was less support among the interviewees for reconsidering the marketing authorization itself (only seven participants saw this as a realistic possibility), mainly because they did not want to restrict the access of patients to innovative therapies if there are no immediate safety-related reasons for doing so, regardless of what is observed during applied clinical research. A withdrawal of the marketing authorization is not required as clinicians would look at reports and publications following from these studies and decide for themselves whether a specific patient should be given a certain treatment, three interviewees remarked.

## Discussion

This study provides a first indication of the perspectives of different drug development stakeholders on the concept of treatment optimization. The results we have obtained may be taken into account when planning and designing trials for the purpose of optimizing the use of novel health technologies in real-world clinical practice (**Table 4**). The views of the interviewed experts align strongly with the sentiments expressed by authors in the field and confirm many of the problems described in the literature. For instance, the current drug development framework has indeed been described as insufficiently patient-centered (Mullins et al., 2014; Ioannidis, 2016; Lacombe et al., 2019b; Wieseler et al., 2019) and unable to produce fit-for-purpose RWE (Kempf et al., 2017). Nevertheless, a notable discrepancy between our findings and the strategies proposed by Lacombe et al. (2019b) is that most of the interviewees who explicitly favored a particular setting in which such research could take place were reluctant to endorse the international approach advocated by the latter. Additionally, while Lacombe et al. (2019b) only mention public funding as a potential mechanism for financing the conduct of treatment optimization studies, the majority of participants saw combinations of public and private funding as a more realistic and appropriate solution. These divergent beliefs however do not detract from the core message that there is a substantial and tangible need for applied clinical research to fill the evidence gap and that considerable support exists for its implementation into the current framework for developing new medicines.

**Table 4:** Summary of the ideal features of treatment optimization studies according to the experts interviewed.

Feature	Findings from interviews
Conduct	Consortia comprised of all relevant stakeholders OR Academia and not-for-profit organizations, with support from industry (drug supply)
Funding	Combinations of public and private funding
Timing	No clear consensus whether pre- or post-approval
Design	Fewer inclusion and exclusion criteria Standard of care or best available treatment as comparators Patient-relevant outcome measures No clear consensus on blinding No clear consensus on randomization Publication of all results
Setting	No particular preference, decided on case-by-case basis OR National, with international coordination and oversight

Such a shift in the prevailing drug-centered paradigm can only be achieved through the creation of a set of concrete policy proposals. The EORTC has composed a manifesto (EORTC, 2019) in which several directions for changes and policy actions are outlined that could help establish treatment optimization as an essential element within the development pathway of personalized medicine in Europe. This call to action was officially presented during a workshop organized by the Panel for the Future of Science and Technology (STOA) at the European Parliament in Brussels (European Parliament STOA, 2019) and has received support from a multitude of different stakeholders, including scientific societies, patient organizations, industry associations, and Members of the European Parliament. The results that are presented in this paper corroborate many of the points raised in the manifesto, although the optimal timing, funding sources, and setting of treatment optimization studies remain points of contention.

The EORTC manifesto asserts that treatment optimization should be introduced as a formal and obligatory step in every new health technology’s path to market access (EORTC, 2019). Three potential strategies to achieve this emerged from the interviews. While all of them involve utilizing the ability of regulators or payers to demand additional evidence from manufacturers to strengthen their decision-making, they mainly differ in terms of the timing of the trials in question as well as the regulatory mechanism through which such studies would be solicited.

The first strategy, in which data derived from applied clinical research would become part of the collection of evidence that the pharmaceutical industry has to submit to EMA in order to obtain a marketing authorization for their products (Scholz, 2015), requires the manufacturers to engage in treatment optimization early on, before crucial milestones on the road to market access are reached. This would allow its findings to be taken into account during the decision-making process of both EMA and the national payer authorities. Consequently, by raising the evidentiary bar, there would be a lower risk of taking up ineffective or inadequately characterized therapies into clinical practice (Prasad and Cifu, 2011). However, there are also disadvantages associated with this approach. Firstly, the industry would have to invest additional time and costs to meet the increased burden of proof placed upon them (Naci et al., 2012), which could lower their overall productivity (Ruffolo, 2006; Paul et al., 2010) and R&D efficiency (Scannell et al., 2012), and may be used as a valid argument to increase the prices of their products when they eventually enter the market (Barton and Emanuel, 2005; Moors et al., 2014). Secondly, it could extend the duration of the medicines development process, thereby delaying patient and clinician access to innovative treatments (Eichler et al., 2008). Many interviewees perceived this as an undesirable outcome that should be avoided as much as possible. Thirdly, as the pre-approval research setting is largely coordinated by the manufacturer, it would be difficult to maintain and safeguard the independent conduct of such treatment optimization studies, potentially giving rise to the introduction of bias into their design (Sismondo, 2008; Lexchin, 2012), especially when commercial interests are at stake (e.g. in case of decreased therapeutic duration or lower optimal dosing). Neither the interviewees nor authors in the field (Angell, 2004; Lewis et al., 2007; Ioannidis, 2013; Kempf et al., 2017; Lacombe et al., 2019b) advocate such an industry-driven approach, instead preferring that academic institutions, not-for-profit organizations, and/or governmental bodies would be given a more prominent role in clinical research in general or treatment optimization in particular.

In the second strategy, the conduct of treatment optimization studies would be implemented into EMA’s conditional approval procedure (EMA, 2016b; EMA, 2017) as a post-authorization commitment to be fulfilled by the manufacturer. A major advantage of this approach, if properly organized, is that it does not impede patient and clinician access to new therapies. Once the initial marketing authorization has been awarded and the national reimbursement process has been successfully completed, the treatment will be available for use in clinical practice in that particular country. In the meantime, the manufacturer could deliver on their applied clinical research obligations, for example by setting up partnerships with independent research institutions. Additionally, to counter the argument of increased development costs, the revenue generated by the company from the sale of their product during this period could be partially used to finance these trials, possibly in combination with public funding. Moreover, since the original approval will be revisited after a certain amount of time, the results of these studies would have a direct impact on regulatory decision-making. This strategy is also in line with the adaptive pathways model (Eichler et al., 2015; EMA, 2016a) designed by EMA. Nevertheless, while the utilization of the conditional approval mechanism is currently restricted to treatments addressing an unmet medical need (EMA, 2016b; EMA, 2017), treatment optimization will likely also be necessary in areas where alternative therapeutic strategies already exist, such as in the context of trials investigating combinations or comparisons of different therapies. In addition, the conditional approval procedure as it is applied today allows the industry to launch new medicines into the market based on incomplete datasets (e.g. data from phase II trials) (EMA, 2016b; EMA, 2017), thereby possibly contributing to the widening of the research gap (Gellad and Kesselheim, 2017; Gyawali et al., 2019; Schuster Bruce et al., 2019). Care should be taken that in our efforts to establish the treatment optimization concept, we do not inadvertently magnify the underlying problem.

The third strategy would incorporate applied clinical research into the conditional reimbursement procedures coordinated by the national payer authorities. The appropriate legal tools to effectuate such an approach exist already and are typically referred to as managed entry agreements (MEAs) (Ferrario and Kanavos, 2013; Gerkens et al., 2017). These are contractual arrangements between a pharmaceutical company that has been granted a marketing authorization for a specific health technology and the healthcare payers of a particular country in which the latter can decide to partially cover the costs of the therapy for a predetermined amount of time. At the end of that period, the request for reimbursement is re-evaluated based on new information that has since become available and if appropriate, a more permanent coverage plan can be set up. However, depending on the results of this assessment, the agreement can also be prolonged or voided. Multiple different types of MEAs can be distinguished. For the purpose of this strategy, the outcome-based MEAs, which establish a link between the reimbursement of a drug and its effects on outcomes in real-world clinical practice (Ferrario and Kanavos, 2013; Gerkens et al., 2017) are especially relevant. More specifically, coverage with evidence development (CED) schemes could be used as a vehicle to implement treatment optimization. In this subtype of outcome-based MEA, the manufacturer commits to gathering the necessary supplementary evidence to convince the payers that their product should receive permanent reimbursement (Carlson et al., 2010; Ferrario and Kanavos, 2013; Gerkens et al., 2017). Applied clinical research could potentially be integrated into CED schemes and produce this additional proof demanded by the payers.

Advantages of this third strategy include that it does not impair access of patients and clinicians to promising new therapies (Russo et al., 2010; Towse, 2010; Brugger et al., 2015; Gerkens et al., 2017) and that it allows the results of treatment optimization studies to be taken into consideration during the decision-making of payers. Moreover, instead of depending on EMA to scrutinize the data obtained from such trials, this responsibility is transferred to the payers, who traditionally review information on the effectiveness of health technologies in real-life patients and can rely on the expert advice of HTA agencies to support them in their assessments. Additionally, for the same reason as the previous strategy, the issue of increased development costs is less pertinent here, although finding a sustainable funding mechanism for applied clinical research remains a challenge. A post-approval setting also offers more opportunity for collaboration with independent research institutions. Nevertheless, there are several disadvantages accompanying this approach. For example, the laws governing the use of MEAs are encoded in national legislation (Gerkens et al., 2017), meaning that there will be differences between European countries with respect to the circumstances under which these schemes may be applied (de Pouvourville, 2006; Towse and Garrison, 2010). Therefore, it would be much more challenging to perform and coordinate treatment optimization studies in an international setting, since each EU Member State has different priorities and thus could ask for divergent research questions or topics to be incorporated. In addition, only a small minority of MEAs currently in place are outcome-based (Carlson et al., 2014; Macaulay and Jamali, 2015; Gerkens et al., 2017), partly because this type of conventions presents a significant financial and administrative burden for the parties involved (Carlson et al., 2009; Adamski et al., 2010; Neumann et al., 2011; Gerkens et al., 2017). It remains to be seen whether the volume of treatment optimization studies needed would exceed payers’ capacity to follow up on their outcomes effectively, and if so, how it should be decided which research questions to prioritize. Furthermore, in past examples of outcome-based MEAs as well as MEAs in general, the conditions for coverage often eventually remained unchanged, even when insufficient evidence was provided by the manufacturer at the end of the initial period of reimbursement (Mortimer et al., 2011; Bishop and Lexchin, 2013; Lewis et al., 2015; Gerkens et al., 2017). It would seem that payers are reluctant to act upon the data collected in such schemes, in part due to ethical objections surrounding the cessation of reimbursement, which defeats the purpose of organizing them in the first place. Applied clinical research should be able to lead to changes in the way health technologies are utilized in clinical practice.

The second and third strategies are also suggested by Lacombe and colleagues (2019a). These approaches strike a good balance between the need of HTA agencies, healthcare payers and clinicians for additional evidence to inform their decision-making and the demands of patients for timely access to innovative therapies. They might therefore be the most pragmatic policy options for introducing treatment optimization into the present drug development framework. Nevertheless, they place the full responsibility of ensuring that applied clinical research data is collected on the industry, which may not be a constructive and sustainable way forward. New mechanisms to stimulate and foster multi-stakeholder evidence generation are needed. Moreover, to implement any of the abovementioned strategies, political support for such a paradigm shift will be required. The European Commission should work together with the Member States to establish treatment optimization as an official step in the path to market access of novel treatments in order to help bridge the research gap and safeguard the sustainability of our healthcare systems.

Our study suffers from three main limitations. Firstly, due to the large number of targeted stakeholder groups, the interviews could not be conducted until data saturation was reached for each individual group. Nevertheless, the multi-stakeholder approach enabled a diverse range of opinions to be captured. Secondly, to accelerate and facilitate the recruitment process, we did not apply a stratified sampling method. Consequently, our findings should not be readily extrapolated to European drug development actors in general. For example, the expertise of the interviewees was limited to oncology, hematology, rheumatology, or pneumology, so their insights and remarks may not be as relevant for other areas of research. Additionally, Western Europe was overrepresented in the list of included EU Member States, which could indicate that the participants’ observations and answers may not be reflective of the situation in other European regions. Thirdly, as a result of time-related and logistical constraints, the analysis of the interview data was performed by a single person as opposed to multiple researchers in parallel, meaning there was no opportunity to validate the coded transcripts by carrying out cross-checks as prescribed by Gale et al. (2013). Despite this, any uncertainties that emerged during the coding process were discussed with one of the principal investigators.

## Conclusion

The current framework for developing new health technologies does not adequately address patient-relevant research questions, leading to the emergence of an evidence gap which severely complicates the decision-making of HTA bodies, payers, and clinicians. Treatment optimization studies could potentially help fill this gap by investigating how novel medicines should be applied in real-world practice so as to optimize their clinical utility. Various stakeholders involved in the drug development process confirmed the need for these organized data collection schemes and shared their views on the optimal features of such trials in terms of their conduct, funding, timing, design, and setting. Furthermore, they identified several opportunities and challenges associated with these studies and considered their evidence strength to be of a sufficiently high level to inform decision-making. They also strongly supported their implementation into the existing clinical research paradigm and suggested three possible mechanisms through which this may be realized. The interviewees’ thoughts and opinions broadly aligned with those expressed by authors in the field. In order for treatment optimization to become a formal prerequisite for adopting therapeutic interventions into our healthcare systems, political support for such a paradigm shift will be required.

## Data Availability Statement

The datasets generated for this study are available on request to the corresponding author.

## Ethics Statement

Ethical review and approval was not required for the study on human participants in accordance with the local legislation and institutional requirements. The patients/participants provided their written informed consent to participate in this study. Written informed consent was obtained from the individual(s) for the publication of any potentially identifiable images or data included in this article.

## Author Contributions

All authors contributed to the conception and design of the study. RS was responsible for collecting, analyzing, and interpreting the data. All authors were involved in drafting and revising the study manuscript and approved of the submitted version.

## Funding

This research was funded by the Panel for the Future of Science and Technology of the European Parliament and the EORTC Cancer Research Fund.

## Disclaimer

The views expressed in this publication are the sole responsibility of the authors and do not necessarily reflect the views of the affiliated organizations.

## Conflict of Interest

The authors declare that the research was conducted in the absence of any commercial or financial relationships that could be construed as a potential conflict of interest.

The reviewer AB declared a past co-authorship with one of the authors, IH, to the handling editor.
