# Supplementary material for: Views of European Drug Development Stakeholders on Treatment Optimization and Its Potential for Use in Decision-Making
Source: Front Pharmacol. 2020 Feb 5;11:43. doi: 10.3389/fphar.2020.00043 (PMC7015135; doi:10.3389/fphar.2020.00043)
Supplement: Supplementary file 1 [file DataSheet_1.docx]

**Supplementary materials**

**Interview questions**

The interview questions were categorized according to three main themes:

*Theme 1: Current situation in drug development research*

1. What do you think of the notion that current drug development research is too drug-centered and not sufficiently patient-centered?
2. How does the current approach impact patients?

(for the patient organisation representatives)

How does the current approach impact clinical decision-making?

(for the academic clinicians)

How does the current approach impact HTA decision-making?

(for the HTA agency representatives)

How does the current approach impact reimbursement-related decision-making?

(for the payers)

How does the current approach impact regulator decision-making?

(for the regulators)

How does the industry justify the current approach they are taking?

(for the industry representatives)

1. What is your opinion regarding the statement that there is a lack of real-world evidence for the use of many drugs on the market today?

What are some of the reasons, you think, for this lack of real-world evidence?

(if agreement was expressed with the statement)

*Theme 2: Features of treatment optimization studies*

1. Who should perform treatment optimization studies (industry, academia, not-for-profit organisations,…)?
2. How should these studies be funded (public funding, private funding, combinations of the two,…)?
3. When should these studies best take place in the drug development process?
4. What would be some of the most important features that treatment optimization studies should have (in terms of objectives, recruitment, randomization, blinding, follow-up, outcomes, reporting, etc.) to be as relevant as possible for clinical practice?
5. What would be the best setting for the conduct of these studies (local, national, international)?
6. How would members of your stakeholder group react if they were invited to contribute to the planning and conduct of treatment optimization studies?
7. What could members of your stakeholder group bring to the table?
8. Which other stakeholders should be at the table?

*Theme 3: Acceptability of treatment optimization studies*

1. What is your opinion on the assertion that regulatory agencies should take measures to facilitate treatment optimization?

What kind of measures should be taken?

(if agreement was expressed with the assertion)

1. What are some of the advantages/opportunities you can think of as a member of your stakeholder group with respect to the conduct of these treatment optimization studies?
2. What are some of the disadvantages/challenges you can think of as a member of your stakeholder group with respect to the conduct of these treatment optimization studies?
3. How would you view the evidence strength of these studies in the context of making treatment decisions for patients?

(for the patient organisation representatives and academic clinicians)

How would you view the evidence strength of these studies in the context of making HTA decisions?

(for the HTA agency representatives)

How would you view the evidence strength of these studies in the context of making reimbursement-related decisions?

(for the payers)

How would you view the evidence strength of these studies in the context of making decisions to approve drugs for use in patients?

(for the regulators)

How would you view the evidence strength of these studies in the context of decision-making by regulators and reimbursement legislators?

(for the industry representatives)

1. Do you have any additional remarks, questions, doubts, concerns regarding the topic of treatment optimization?

Note that statements extracted from the literature served as the basis for questions 1 (Lacombe et al., 2019b), 3 (Kempf et al., 2017) and 12 (EORTC, 2019; Kempf et al., 2017; Lacombe et al., 2019a).
